# Supplementary material for: Machine learning-based drought prediction using Palmer Drought Severity Index and TerraClimate data in Ethiopia
Source: PLoS One. 2025 Jun 18;20(6):e0326174. doi: 10.1371/journal.pone.0326174 (PMC12176240; doi:10.1371/journal.pone.0326174)
Supplement: Attachment — Submitted filename: PONE-D-24-44104_Rewview comments.docx [file pone.0326174.s001.docx]

**Review Comments**

Here is a detailed review of research article, "Machine Learning-Driven Drought Prediction Using Palmer Drought Severity Index and Terra Climate Data over Ethiopia."

**Overall Assessment**

The study is well-structured and presents a significant contribution to drought prediction using machine learning. The use of multiple classifiers and evaluation metrics provides a comprehensive analysis. However, there are a few areas where improvements could enhance the clarity, robustness, and impact of the study.

**Major Comments**

**Justification for Model Selection**

- The study uses multiple machine learning models, but a stronger justification for selecting these specific models (e.g., Logistic Regression, Random Forest, SVM, KNN, etc.) should be included.
- Consider briefly discussing why deep learning models (CNN, LSTM) were not included, given their growing use in environmental modeling.

**Handling of Imbalanced Data**

- The manuscript mentions using SMOTE and manual duplication to address data imbalance, but this method might lead to artificial patterns in the dataset.
- A comparison with other balancing techniques (such as class-weighting or cost-sensitive learning) would be beneficial.

**Feature Importance Analysis**

- The study mentions that key drought predictors such as temperature and precipitation were not always ranked highly. This contradicts conventional knowledge and should be explained more clearly.
- Conducting SHAP (SHapley Additive Explanations) analysis could offer deeper insights into feature contributions.

**Uncertainty and Limitations**

- There is little discussion on the uncertainties associated with machine learning predictions, including possible biases in the TerraClimate dataset.
- A sensitivity analysis of input variables could improve confidence in the model outputs.

**Comparison with Traditional Models**

- Since traditional statistical models (e.g., autoregressive models, time-series forecasting) are commonly used in drought prediction, it would strengthen the paper to compare at least one traditional model with the machine learning models.

**Minor Comments**

1. **Clarity in Model Performance Discussion**

- The discussion on Random Forest and Gradient Boosting being the best models should explicitly state their precision, recall, and F1-score rather than focusing mainly on accuracy and ROC-AUC.

2. **Data Preprocessing Details**

- More details on how missing data was handled (linear interpolation was mentioned, but were other methods like KNN imputation tested?) would strengthen the methodology.

3. **Figures & Tables**

- Ensure that all figures are high resolution and clearly labeled (some descriptions refer to figures without clear reference in the text).
- Consider adding a table comparing model performance metrics for quick readability.

4. **Language & Grammar**

- Minor grammatical errors and awkward phrasing exist (e.g., “The dataset shows varying degrees of drought and wet conditions, with Near Normal being the most common category” → consider rewording for clarity).
- Proofreading for conciseness and academic tone is recommended.
