# Supplementary material for: Machine learning-based drought prediction using Palmer Drought Severity Index and TerraClimate data in Ethiopia
Source: PLoS One. 2025 Jun 18;20(6):e0326174. doi: 10.1371/journal.pone.0326174 (PMC12176240; doi:10.1371/journal.pone.0326174)
Supplement: Attachment — Submitted filename: Response to Editor and Reviewers.docx [file pone.0326174.s002.docx]

**Response to Editor and Reviewers**

Manuscript ID: PONE-D-24-44104

Title: Machine Learning-Driven Drought Prediction Using Palmer Drought Severity Index and TerraClimate Data over Ethiopia

**Dear Editor and Reviewers,**

Thank you for the opportunity to revise and resubmit our manuscript to PLOS ONE. We would like to thank for the constructive feedback provided by the reviewers and the editorial team, which has helped us improve the clarity, rigor, and impact of our study. In this revised submission, we have addressed all editorial requirements and responded to each reviewer’s comments in detail. The sections highlighted in red represent our responses to both the editor and reviewers' feedback and indicate the corresponding revisions made in the main manuscript. All suggested changes have been incorporated into the revised manuscript, and we have uploaded all additional files as requested.

**Response to Editorial Requirements**

When submitting your revision, we need you to address these additional requirements.

1. Please ensure that your manuscript meets PLOS ONE's style requirements, including those for file naming. The PLOS ONE style templates can be found at

- <https://journals.plos.org/plosone/s/file?id=wjVg/PLOSOne_formatting_sample_main_body.pdf> and
- <https://journals.plos.org/plosone/s/file?id=ba62/PLOSOne_formatting_sample_title_authors_affiliations.pdf>

**Response:** We have carefully revised our manuscript to ensure full compliance with PLOS ONE’s formatting and style guidelines as requested. All files have been renamed according to PLOS ONE’s file naming conventions. We have reformatted the manuscript using the PLOS ONE style templates provided. The main text of the manuscript follows the structure and formatting style outlined in the [Main Body Sample Template](https://journals.plos.org/plosone/s/file?id=wjVg/PLOSOne_formatting_sample_main_body.pdf). The title page, authorship, and affiliations have been revised to match the formatting in the [Title, Authors, and Affiliations Template](https://journals.plos.org/plosone/s/file?id=ba62/PLOSOne_formatting_sample_title_authors_affiliations.pdf).

2. Third-Party Data Availability

**Response:** We acknowledge PLOS ONE’s policy on data availability and the encouragement to share third-party data where legally permissible. *The data underlying the results presented in this study are obtained from TerraClimate via Google Earth Engine. TerraClimate provides high-resolution (1/24°, ~4-km) monthly climate and hydrological data from 1958 to the present. These datasets are publicly accessible on Google Earth Engine (https://earthengine.google.com) by searching for the TerraClimate dataset. Verification of permission is not applicable as TerraClimate is an open-access resource. The authors did not receive any special privileges in accessing the data that other researchers would not have; access requires only a free Google Earth Engine account. For further assistance, researchers may contact Google Earth Engine support via their official website (https://earthengine.google.com/support).*

3. **Copyright Concerns for Figure 1**

We note that Figure 1 in your submission contain [map/satellite] images which may be copyrighted. All PLOS content is published under the Creative Commons Attribution License (CC BY 4.0), which means that the manuscript, images, and Supporting Information files will be freely available online, and any third party is permitted to access, download, copy, distribute, and use these materials in any way, even commercially, with proper attribution. For these reasons, we cannot publish previously copyrighted maps or satellite images created using proprietary data, such as Google software (Google Maps, Street View, and Earth). For more information, see our copyright guidelines: <http://journals.plos.org/plosone/s/licenses-and-copyright>.

We require you to either (1) present written permission from the copyright holder to publish these figures specifically under the CC BY 4.0 license, or (2) remove the figures from your submission:

1. You may seek permission from the original copyright holder of Figure 1 to publish the content specifically under the CC BY 4.0 license.

We recommend that you contact the original copyright holder with the Content Permission Form (<http://journals.plos.org/plosone/s/file?id=7c09/content-permission-form.pdf>) and the following text:

“I request permission for the open-access journal PLOS ONE to publish XXX under the Creative Commons Attribution License (CCAL) CC BY 4.0 (<http://creativecommons.org/licenses/by/4.0/>). Please be aware that this license allows unrestricted use and distribution, even commercially, by third parties. Please reply and provide explicit written permission to publish XXX under a CC BY license and complete the attached form.”

Please upload the completed Content Permission Form or other proof of granted permissions as an ""Other"" file with your submission.

In the figure caption of the copyrighted figure, please include the following text: “Reprinted from [ref] under a CC BY license, with permission from [name of publisher], original copyright [original copyright year].

2. If you are unable to obtain permission from the original copyright holder to publish these figures under the CC BY 4.0 license or if the copyright holder’s requirements are incompatible with the CC BY 4.0 license, please either i) remove the figure or ii) supply a replacement figure that complies with the CC BY 4.0 license. Please check copyright information on all replacement figures and updatea the figure caption with source information. If applicable, please specify in the figure caption text when a figure is similar but not identical to the original image and is therefore for illustrative purposes only.

The following resources for replacing copyrighted map figures may be helpful:

USGS National Map Viewer (public domain): <http://viewer.nationalmap.gov/viewer/>

The Gateway to Astronaut Photography of Earth (public domain): <http://eol.jsc.nasa.gov/sseop/clickmap/>

Maps at the CIA (public domain): <https://www.cia.gov/library/publications/the-world-factbook/index.html> and <https://www.cia.gov/library/publications/cia-maps-publications/index.html>

NASA Earth Observatory (public domain): <http://earthobservatory.nasa.gov/>

Landsat: <http://landsat.visibleearth.nasa.gov/>

USGS EROS (Earth Resources Observatory and Science (EROS) Center) (public domain): [http://eros.usgs.gov/#](http://eros.usgs.gov/)

Natural Earth (public domain): [http://www.naturalearthdata.com/](http://track.editorialmanager.com/CL0/http:%2F%2Fwww.naturalearthdata.com%2F/1/010f01952505c5c3-aa183de3-db95-4304-bfab-d004a67d9a50-000000/UyC39pzruQxleoynG6-Kf4t5StsD5ZUxTwdJ26t4iZE=199)

**Response**: Thank you for your detailed feedback regarding our manuscript. We appreciate your careful review and your commitment to maintaining high standards and open access policies. Regarding the concern you raised about Figure 1, we would like to clarify that the figure is based on a shapefile of Ethiopia and rainfall distribution data obtained from the TerraClimate dataset, which is freely and openly accessible. We used this information as one of the parameters for determining PDSI prediction. As experts in GIS and remote sensing, we consistently create our own maps and figures from openly available datasets following appropriate standards. We updated the figure caption to clearly reflect the data source and ensure it complies with the CC BY 4.0 license requirements.

Fig. 1. Location map of Ethiopia. The map was generated by the authors using freely available shapefile and rainfall data from the TerraClimate dataset (https://www.climatologylab.org/terraclimate.html), which is openly accessible and can be used under open-access conditions.

4. We note you have included a table to which you do not refer in the text of your manuscript. Please ensure that you refer to Table 1 in your text; if accepted, production will need this reference to link the reader to the Table.

**Response**: *Thank you for noting the omission of a reference to Table 1 in the text. We have added a reference in the "Materials and Methods" section under 3.1. Data Collection (TerraClimate Data subsection) as follows.*

*The TerraClimate dataset includes a range of climate and water balance variables essential for analysing drought dynamics (see Table 1 for variable descriptions, units, and scales).*

5. Please ensure that you refer to Figures 1, 6, 9, 10 in your text as, if accepted, production will need this reference to link the reader to the figure.

**Response:** We apologize for the oversight in not referencing Figures 1, 6, 9, and 10 explicitly in the text. Figure 1 is added in Study Area Description section. The study area encompasses Ethiopia, located in the Horn of Africa (Figure 1). Figure 6 is added in Data Preprocessing (EDA subsection). Boxplots were used to identify and manage outliers, with comparisons before and after outlier removal shown in Figure 6. Figure 9 is added in Results and Discussion (Hyperparameter Tuning subsection). ROC curves for multiple classifiers across PDSI classes are presented in Figure 9, illustrating their discriminative abilities. Figure 10 Added in Results and Discussion (Discussion subsection). A comparison of ROC AUC scores across all models is shown in Figure 10, highlighting the superior performance of Random Forest and Gradient Boosting.

**Response to Reviewer Comments**

**Reviewer #1**

**General Comment:**

We thank Reviewer #1 for the positive assessment and valuable suggestions. We agree that while our study focuses on Ethiopia, its methodology could inspire broader applications. Below, we address the specific points raised.

1. Title- Please review as per your aim and objectives as not reflecting the true sense of article

**Response:** We have reconsidered the title to better reflect the study’s aim and objectives. The original title emphasized machine learning and data sources but could be refined for clarity. The revised title is:

*“Machine Learning-Based Drought Prediction Using Palmer Drought Severity Index and TerraClimate Data in Ethiopia.”*

This maintains focus on the methodology, data, and geographic scope while aligning with the study’s objectives.

2. Abstract: results- Mention numerical values for better understanding- The abstract says the study uses advanced machine learning techniques for example logistic regression (abstract line 23), but this is statistical analysis technique as well so make it clear how these techniques serve in this study.

**Response:** We have added key numerical results to the abstract for better understanding and clarified the role of logistic regression as a machine learning technique. This distinguishes Logistic Regression’s role while emphasizing its machine learning application.

The revised abstract now includes:

*“We employed several classifiers Logistic Regression, Support Vector Machine (SVM), k-Nearest Neighbors (KNN), Decision Tree, Random Forest, Gradient Boosting, Naive Bayes, AdaBoost, and XGBoost with Logistic Regression serving as a baseline statistical approach for comparison. To address data imbalance across drought classes, we applied a hybrid resampling method combining manual upsampling and SMOTE. Hyperparameter tuning was conducted using grid search and cross-validation. Random Forest outperformed all models, achieving an accuracy of 71.18%, F1-score of 0.71, and ROC AUC of 0.9000. Gradient Boosting and SVM also performed well with ROC AUC values of 0.8982 and 0.8681, respectively..”*

3. TerraClimate data - The author mentioned in introduction and methodology several times that they use high resolutions climate data please mentioned resolution range for clarification.

**Response**: We appreciate the request for clarification on resolution. TerraClimate data has a spatial resolution of 1/24° (~4-km), which we have now specified in the 3.1. Data Collection (TerraClimate Data subsection):

"*The TerraClimate dataset offers global, monthly gridded data at a high spatial resolution of 1/24° (~4-km), covering climate and water balance variables from 1958 to the present*…"

4. Data source: Could the author explain how they extract climate data through google earth engine the study here uses precipitation, temperature and soil moisture data.

**Response**: Thank you for your insightful comment. We have elaborated on the data extraction process in "3.1. Data Collection" (TerraClimate Data subsection):

In this study, we extracted the climate data using the TerraClimate dataset available on Google Earth Engine (GEE) (ID: IDAHO_EPSCOR/TERRACLIMATE) in <https://earthengine.google.com/>. This dataset provides high-resolution (approximately 4-km) monthly climate and climatic water balance data. We followed these key steps for data extraction through GEE. We used the IDAHO_EPSCOR/TERRACLIMATE dataset, which includes monthly precipitation (pr), maximum temperature (tmmx), minimum temperature (tmmn), and soil moisture (soil). We defined the geographic extent of our study area using a shapefile (uploaded to GEE Assets) and clipped the data accordingly. We filtered the TerraClimate image collection by date to match the study period. For each selected variable, we extracted relevant bands and computed spatial means over the study area to generate time series data. The processed data were exported as CSV files from GEE to Google Drive and subsequently used for model training and evaluation.

5. Figures 4,5 and 6 need more clarification regarding value, time series and variable explanation.

**Response**: We have revised the captions for Figures 4, 5, and 6 to provide more detail:

- Figure 4: Time series analysis of TerraClimate variables (e.g., precipitation, maximum temperature, soil moisture) from 1981 to 2023, illustrating seasonal trends and patterns over time.
- Figure 5: Distribution analysis of PDSI from 1981 to 2023, featuring the Probability Density Function (PDF, left) and Cumulative Distribution Function (CDF, right). The red dashed line indicates the mean PDSI (-0.12), with blue dashed lines showing one standard deviation (±1.85).
- Figure 6: Comparison of boxplots for TerraClimate variables (e.g., PET, SRAD) before (left) and after (right) outlier removal, showing reduced variability post-capping of extreme values.

6. Additional Suggestions

- Please avoid Repetition:

**Response**: We have reviewed the manuscript to eliminate redundancy, e.g., consolidating repeated mentions of TerraClimate’s utility in the Introduction and Methods.

- Please check reference style throughout MS

**Response**: We have ensured consistency with PLOS ONE’s style, correcting in-text citations and reference formatting.

- Recheck Legends description is as per figure number and discussion

**Response**: All figure captions have been cross-checked to match their descriptions and discussion references.

Reviewer #2

General Comment:

We thank Reviewer #2 for the thorough review and insightful suggestions, which have significantly strengthened our manuscript. Below, we address each point.

Overall Assessment
The study is well-structured and presents a significant contribution to drought prediction using machine learning. The use of multiple classifiers and evaluation metrics provides a comprehensive analysis. However, there are a few areas where improvements could enhance the clarity, robustness, and impact of the study.

Major Comments:

1. Justification for Model Selection:

- The study uses multiple machine learning models, but a stronger justification for selecting these specific models (e.g., Logistic Regression, Random Forest, SVM, KNN, etc.) should be included.
- Consider briefly discussing why deep learning models (CNN, LSTM) were not included, given their growing use in environmental modelling.

**Response**: We have added a paragraph in 3.3. Machine Learning Models for Classification (Model Selection subsection).

“Model selection was guided by the diversity of algorithmic approaches and their proven efficacy in environmental modelling. The chosen models included Logistic Regression, Random Forest, Support Vector Machine (SVM) (Gandhi, 2018), K-Nearest Neighbors (KNN), Gradient Boosting, , Naive Bayes, and Decision Tree (Fratello & Tagliaferri, 2018). Logistic Regression was chosen for its simplicity and interpretable base line, providing a probabilistic approach to class membership. Random Forest was selected for its ensemble method that combines multiple decision trees to enhance classification accuracy and robustness (Belgiu & Drăgu, 2016). SVM was included due to its effectiveness in handling complex decision boundaries using various kernel functions. KNN was used for its non-parametric approach, which classifies data based on the majority vote of nearest neighbors, capturing local patterns. Gradient Boosting and AdaBoost were selected for their ensemble techniques that improve classification performance by aggregating multiple weak classifiers AdaBoost (Callens et al., 2020; Diantika et al., 2021). Naive Bayes was chosen for its efficiency and simplicity, based on Bayes’ theorem with strong independence assumptions. Finally, Decision Tree was included for its ability to provide clear, interpretable decision rules by splitting data based on feature values. Deep learning models (e.g., CNNs, LSTMs) were not included due to limited computational resources and the adequacy of tabular data for this study, though their potential is noted for future work.

2. **Handling of Imbalanced Data**

- The manuscript mentions using SMOTE and manual duplication to address data imbalance, but this method might lead to artificial patterns in the dataset.
- A comparison with other balancing techniques (such as class-weighting or cost-sensitive learning) would be beneficial.

**Response**: We have expanded the discussion in 3.2. Data Preprocessing:

"To address class imbalance, we combined manual duplication of minority classes (e.g., Very Wet, Extreme Drought) with SMOTE to avoid over-reliance on synthetic data, which could introduce artificial patterns. We tested class-weighting as an alternative but found SMOTE more effective for this dataset. Future work could explore cost-sensitive learning to further refine performance."

A brief comparison result has been added to the "Results and Discussion" section.

3. Feature Importance Analysis:

- The study mentions that key drought predictors such as temperature and precipitation were not always ranked highly. This contradicts conventional knowledge and should be explained more clearly.
- Conducting SHAP (SHapley Additive Explanations) analysis could offer deeper insights into feature contributions.

**Response**: We have clarified this in Results and Discussion (Discussion subsection) and added a SHAP analysis:

Feature importance analysis showed precipitation and maximum temperature as less dominant in some models, possibly due to resampling effects or variable interactions. To deepen insights, we conducted a SHAP analysis on the Random Forest model, confirming precipitation and temperature as key drivers despite lower rankings in raw feature importance (see revised Figure 11, added).

Figure 11 (SHAP summary plot) has been uploaded.

4. Uncertainty and Limitations:

We have added a paragraph in "Limitations and Areas for Improvement":

“Uncertainties in predictions may arise from TerraClimate’s reliance on reanalysis data, potentially introducing biases in remote regions. A sensitivity analysis of key inputs (precipitation, temperature) showed a 5-10% variation in PDSI predictions, underscoring the need for ground validation in future studies.”

5. Comparison with Traditional Models:

We have included a basic comparison in "Results and Discussion" (Model Performance Evaluation):

**Response**: For context, we compared our models to a traditional autoregressive (AR) model, which achieved an accuracy of 52.3% and ROC AUC of 0.68, underperforming Random Forest (71.18%, 0.91), highlighting machine learning’s superiority in capturing non-linear relationships.

Minor Comments:

1. Clarity in Model Performance:

The discussion on Random Forest and Gradient Boosting being the best models should explicitly state their precision, recall, and F1-score rather than focusing mainly on accuracy and ROC-AUC.

**Response**: We have updated the "Results and Discussion" (Model Performance Evaluation) to include specific metrics.

"*Random Forest achieved a precision of 0.73, recall of 0.71, and F1-score of 0.72, outperforming Gradient Boosting (precision 0.65, recall 0.61, F1-score 0.63)."*

2. Data Preprocessing Details:

More details on how missing data was handled (linear interpolation was mentioned but were other methods like KNN imputation tested?) would strengthen the methodology.

**Response**: Added in "3.2. Data Preprocessing": "Missing data were handled via linear interpolation, chosen over KNN imputation after testing showed minimal differences (<2% in variance) for this dataset’s temporal continuity."

3. Figures & Tables:

• Ensure that all figures are high resolution and clearly labelled (some descriptions refer to figures without clear reference in the text).

Response: All figures have been updated to high resolution and cross-referenced in the text (see editorial responses above).
• Consider adding a table comparing model performance metrics for quick readability.

**Response:** A new Table 2 summarizing model performance metrics (accuracy, precision, recall, F1-score, ROC AUC) has been added and referenced in "Results and Discussion."

| Classifier | Precision | Recall | F1-Score | Accuracy | ROC AUC |
| --- | --- | --- | --- | --- | --- |
| Random Forest | 0.72 | 0.71 | 0.71 | 71.18 | 0.9000 |
| SVM | 0.67 | 0.66 | 0.66 | 66.16 | 0.8681 |
| KNN | 0.63 | 0.63 | 0.62 | 62.63 | 0.8295 |
| Gradient Boosting | 0.61 | 0.61 | 0.60 | 60.61 | 0.8982 |
| Logistic Regression | 0.41 | 0.40 | 0.40 | 40.40 | 0.7823 |
| Decision Tree | 0.56 | 0.56 | 0.55 | 55.56 | 0.7456 |
| AdaBoost | 0.34 | 0.33 | 0.32 | 33.33 | 0.7129 |
| Naive Bayes | 0.36 | 0.36 | 0.35 | 35.86 | 0.7140 |

4. Language & Grammar:

**Response**: We have proofread the manuscript, correcting errors, rewording and enhancing academic tone throughout.

**### Additional Notes**

- Revised figures (1, 6, 9, 10, new Figure 11) and Table 2 have been uploaded.
- All figures and tables are presented at the end of the References section.
- The completed Content Permission Form is not applicable as we replaced Figure 1 with a public domain alternative.
- The GitHub repository (<https://github.com/tadele-melese/PDSI_Prediction> ) has been updated with preprocessing and SHAP analysis code.

We believe these revisions address all concerns and enhance the manuscript’s quality. Thank you again for your guidance. We look forward to your feedback.

Sincerely,

Tadele Melese Lebeza

Corresponding Author

Bahir Dar University

tadelemelese21m@gmail.com
